# Assessing the impact of a research funder’s recommendation to consider core outcome sets

**DOI:** 10.1371/journal.pone.0222418

**Published:** 2019-09-13

**Authors:** Karen L. Hughes, Jamie J. Kirkham, Mike Clarke, Paula R. Williamson

**Affiliations:** 1 MRC North West Hub for Trials Methodology Research, Department of Biostatistics, University of Liverpool, Liverpool, United Kingdom; 2 Centre for Biostatistics, Manchester Academic Health Science Centre, University of Manchester, Manchester, United Kingdom; 3 Centre for Public Health, Institute of Clinical Sciences, Queen’s University Belfast, Royal Victoria Hospital, Belfast, United Kingdom; Lancaster University, UNITED KINGDOM

## Abstract

**Background:**

Core outcome sets (COS) have the potential to reduce waste in research by improving the consistency of outcomes measured in trials of the same health condition. However, this reduction in waste will only be realised through the uptake of COS by clinical trialists. Without uptake, the continued development of COS that are not implemented may add to waste in research. Funders of clinical trials have the potential to have an impact on COS uptake by recommending their use to those applying for funding. The aim of our study was to assess the extent to which applicants followed the National Institute for Health Research Health Technology Assessment (NIHR HTA) programme’s recommendation to search for a COS to include in their clinical trial.

**Methods and findings:**

We examined the outcomes section and detailed project descriptions of all 95 researcher-led primary research applications submitted to the NIHR HTA between January 2012, when the recommendation to search for a COS was included in the guidance for applicants, and December 2015 for evidence that a search for a COS had taken place and rationale for outcome choice in the absence of COS. A survey of applicants was conducted to further explore their use of COS and choice of outcomes with a response rate of 49%. Nine out of 95 applicants (10%) stated in their application that they had searched the COMET (Core Outcome Measures for Effectiveness Trials) Initiative database for a COS and another nine referred to searching for a COS using another method, e.g. a review of the literature. Of the 77 (81%) applicants that did not mention COMET or COS in their application, eight stated in the survey that they had searched the COMET database and ten carried out a search using another method. Some applicants who did not search for a COS gave reasons for their choice of outcomes including taking advice from patients and the public and choosing outcomes used in previous trials.

**Conclusion:**

A funding body can have an impact on COS uptake by encouraging trialists to search for a COS. Funders could take further steps by putting processes in place to prompt applicants to be explicit about searching for COS in their application and notifying the funding board if a search has not taken place. The sources of information used by trialists to make decisions about outcomes in the absence of COS may suggest methods of dissemination for COS.

## Introduction

Core outcome sets (COS) are agreed sets of outcomes that should be measured and reported, as a minimum, in all clinical trials of the same health condition. They have the potential to reduce waste in research by: improving the consistency of outcomes measured in trials of the same health condition; ensuring that all important outcomes are measured; and reducing outcome reporting bias[1]. However, this reduction in waste will only be realised if COS are actually used by clinical trialists. Indeed, without uptake, the continued development of COS that are not implemented may itself add to research waste by allocating resources to the development of something that is not put into practice.

Several studies have been conducted to assess the uptake of individual COS[2–7]. One such study assessed the uptake of a COS for rheumatoid arthritis up to 15 years after it was published and reported an increasing level of use over time, with almost 70% of trialists measuring the COS at the end of the study period[2]. A subsequent update of this work demonstrated that uptake had continued to increase with 81% of trials in the study period measuring the COS[8]. One factor that may have influenced the uptake of this COS was its endorsement by the Food and Drug Administration in 1996 and European Medicines Agency in 1998, after which an increase in uptake was observed. In contrast, other studies looked at uptake of COS between two and 17 years after publication of the COS[3–7] and reported low uptake; in most cases less than 7% of trials measured all of the outcomes in the COS with none of these studies reporting endorsement by a regulatory body for the particular COS. In order to encourage the uptake of COS by clinical trialists, it is important to investigate potential barriers and facilitators to their use, and doing so may suggest methods of implementation that would maximise uptake.

One strategy to encourage uptake by increasing trialists’ awareness of COS is for funders to recommend their use. In January 2012, the UK National Institute for Health Research Health Technology Assessment Programme (NIHR HTA) added the following statement to its guidance for applicants for all randomised trials and evidence synthesis funding streams:

“Details should include justification of the use of outcome measures where a legitimate choice exists between alternatives.

- Where established Core Outcomes exist they should be included amongst the list of outcomes unless there is good reason to do otherwise. Please see The COMET Initiative website at www.comet-initiative.org to identify whether Core Outcomes have been established.”

The COMET Initiative supports the development and uptake of COS and facilitates this by providing a database of planned, ongoing and completed COS on its website. The database can be searched by trialists to find if a COS exists for the health condition that they will study.

Building from the NIHR HTA’s endorsement, we aimed to investigate its impact, by examining applications to the researcher-led stream of the NIHR HTA programme since 2012 to determine whether trialists followed the guidance to search for a COS using the COMET website, or some other source, and, if so, to find out how they used the information they found, or, if not, to find out how they chose the outcomes for their studies.

## Methods

### Accessing NIHR HTA funding applications for clinical trials

The NIHR Evaluation, Trials and Studies Coordinating Centre, through the Research on Research programme, provided access to data extracted from the outcomes section of all NIHR HTA full primary research applications submitted to the researcher-led funding stream between January 2012 and December 2015 (n = 95). This included funded and non-funded applications and we were also given the detailed project description for each application.

### Extracting the data

The outcomes section of each application form and detailed project descriptions were searched for information included about the COMET website, COS, or other justification of choice of outcomes for the trial. The information was extracted and recorded in a matrix (see S1 Appendix for example of matrix) by the first author (KH). For each application KH also searched the COMET database to establish whether a COS existed at the time of submission, or whether a COS was available that may have been relevant to the application even if not an exact match. Where no COS existed at the time of application the COMET database was searched to determine whether a COS had since been developed. A sample of the database searches were checked for accuracy by PW and JK and any discrepancies were discussed until agreement was reached.

### Survey of Chief Investigators

A survey was then sent to all applicants by email from the NIHR Research on Research team, to further investigate the researcher’s decision to search for and use a COS, or not, and to discover more about their strategies for selecting outcomes. The email contained a link to an online survey set up using SelectSurvey software (https://selectsurvey.net/). One of four versions of the survey was sent to each applicant depending on the information extracted from their application:

1. Survey 1 was sent to applicants who had mentioned the COMET website or COS and had found and used a COS that had been published or was in development.
2. Survey 2 was sent to applicants who had mentioned the COMET website or COS and had not found a relevant COS for their trial.
3. Survey 3 was sent to applicants who had not mentioned the COMET website or COS but had given reasons for their choice of outcomes.
4. Survey 4 was sent to applicants who had not mentioned the COMET website or COS and had not given reasons for their choice of outcomes.

Following the initial mailing follow up emails were sent to non-responders on three occasions. Applicants were asked about their use of the NIHR guidance when completing their application, their decision to search for and use a COS or not, including their assessment of the COS, and reasons for their choice of outcomes where a COS was not found or searched for (see S1 Text for copies of the questions in each of the four surveys).

## Results

### Applications with a COS at the time of submission

A search of the COMET database identified COS for 24 (25%) applications at the time that they were submitted. For three applications the search identified COS that, although not an exact fit, may have been relevant to the health condition in the application. Of the remaining 68 applications that did not have a COS relevant to their health condition at the time, 32 now have COS that have since been developed (as of October 2018).

### Examination of application forms

#### Applicants searching the COMET database

**Fig 1:**
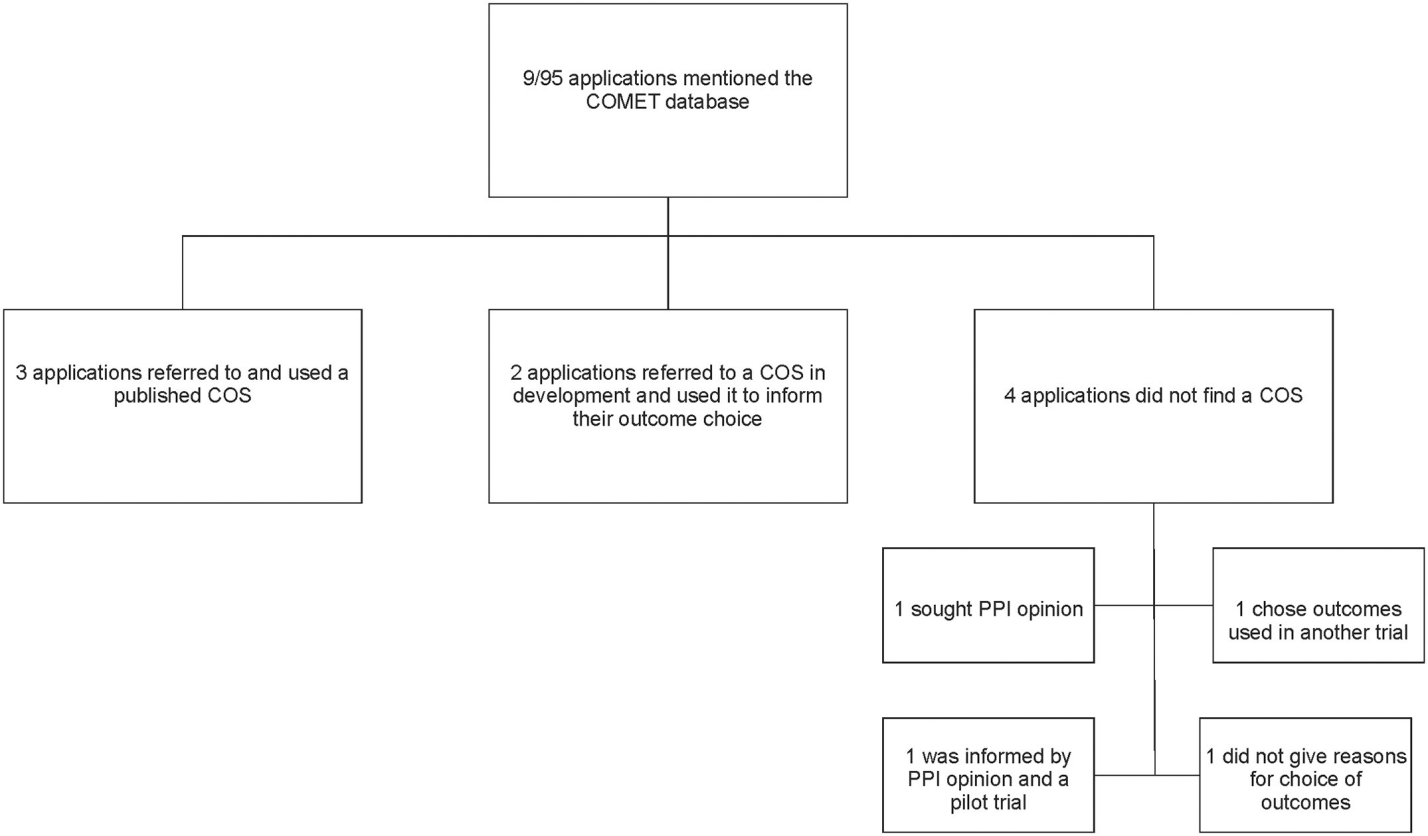
Applications that referred to a search of the COMET database for a COS.

Nine (10%) of the 95 applicants stated in their application that they had searched the COMET database. Three found a relevant published COS and proposed that it would be included in their study, two found a relevant COS that was in development and used the interim findings to inform their decision on which outcomes to include in their study, and four did not find a relevant COS (Fig 1). A search of the COMET database by one author (KH) identified that COS did exist for the conditions being studied in two of these four trials but the applicants stated that their search did not identify relevant COS. Although a COS did not exist for one of the four applications at the time of submission, a COS in development was registered on the COMET website, and for the fourth application there is no COS in the COMET database as of October 2018. Three of the four applicants who searched COMET but did not find a COS relevant to their study explained how they reached the decision about which outcomes to include in their trial. Fig 1 shows that reasons given included the opinion of patients or the public, information from a pilot trial, and the use of outcomes that had been used in previous trials of the same health condition.

#### Applicants referring to a COS

**Fig 2:**
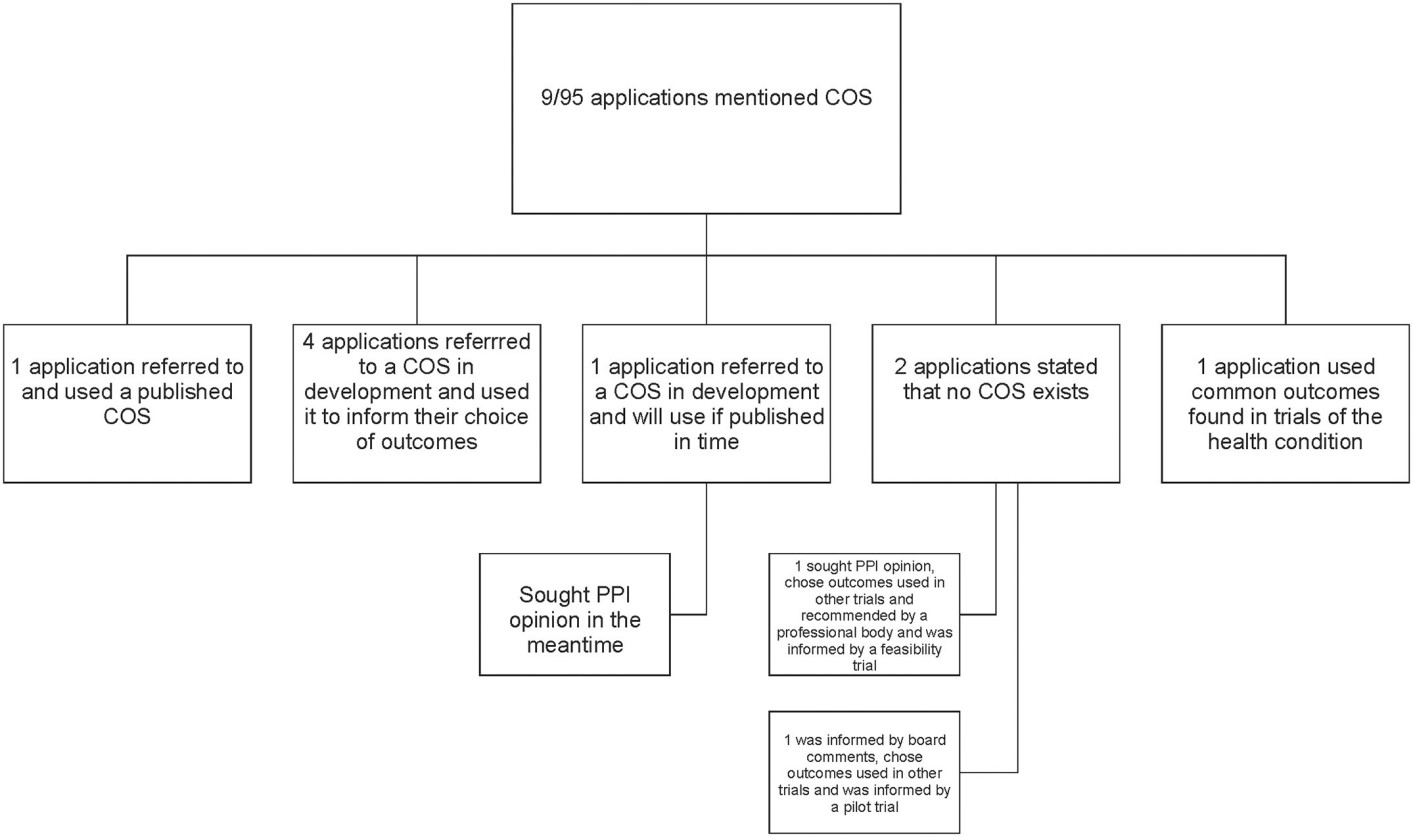
Applications that referred to a COS but did not make reference to the COMET database.

Nine (10%) applicants mentioned COS in their application but did not make reference to the COMET database: one referred to a published COS that they intended to include in their study; five referred to a COS in development (with four of these using the COS to inform their choice of outcomes and the fifth stated that they would use the COS if it was published in time for their study but sought patient opinion in the meantime); two applicants stated that no relevant COS existed but did not explain the steps that they took to find this out; one applicant referred to core outcomes in terms of common outcomes used in trials of their health condition but a COS had not been developed (Fig 2). A search of the COMET database by one author (KH) confirmed that no relevant COS were recorded for the conditions of interest in the two trials that did not find a COS. These applicants used a combination of patient and public opinion, outcomes used in other trials and recommended by a professional body, funding board feedback, and information from a feasibility or pilot trial to inform their choice of outcomes in the absence of COS (Fig 2).

#### Sources of information accessed by trialists to inform their decision about the outcomes to include in their study

Of the remaining 77 applications that did not mention the COMET database or COS, 58 contained information about how the researchers chose the outcomes to include in their study and some common themes were apparent. Examples of the information extracted for each theme are presented in Table 1.

**Table 1:** Examples of the sources applicants used to inform their choice of outcomes as extracted from the applications.

Source	Number (%) of trials mentioning this source	Example
Patient and public opinion	31 (53%)	Feedback from parents led to changes in the outcome measures we will use …
Outcomes used in other trials	22 (38%)	We have selected this measure because of its… properties including …, and because it has been widely used in other randomised trials of… with …
Recommendation from a professional body	13 (22%)	The primary outcome measure is… (as recommended by the… Association for …)
Feedback from the funding board	12 (21%)	The outcomes have been amended taking into account the board’s recommendation …
Information from a feasibility/pilot trial	9 (16%)	… and data from our pilot trial were used to inform choice of outcome measures and the sample size calculations.
Practitioner opinion	3 (5%)	… is the key outcome for clinicians.

**Patient and public opinion**. Thirty one applicants (53%) described seeking the views of patients and the public with knowledge of the condition of interest on the outcomes that had been chosen, or asking for their opinion on which outcomes should be included in the study. In some cases, the researchers described changing or adding outcomes based on public and patient feedback.

**Outcomes used in other trials**. Twenty two applicants (38%) looked at outcomes used in previous trials to inform their choice. One reason given for this was to enable comparison between trials.

**Recommendation from a professional body**. Thirteen applicants (22%) sought information from a professional body associated with the health condition that was the subject of their trial and included an outcome that had been recommended by the professional body.

**Feedback from the funding board**. Twelve applicants (21%) amended at least one of their outcomes following recommendations fed back from the funding board. This included adding outcomes not included in the preliminary application, reducing the number of outcomes included in the preliminary application, and in six of the 12 applications making changes to the primary outcome.

**Information from a feasibility/pilot trial**. Nine applicants (16%) used the results of a feasibility or pilot trial to inform their choice of outcomes. In three cases, the applicants amended their outcomes following the feasibility/pilot trial, for example, by reducing the number of outcomes to reduce patient burden and improve retention.

**Practitioner opinion**. Three applicants (5%) were explicit that they took on board the opinion of practitioners when selecting outcomes, referring to surveys of clinicians either published previously or conducted by the applicant.

#### Applications that did not include reasons for choice of outcomes

Twenty (21%) applicants did not explain how or why they chose the outcomes to include in their proposal. One of these applicants had searched for but did not find a COS and 19 did not state whether they had searched for a COS before choosing outcomes for their study.

### Survey of Chief Investigators

Forty-seven of the 95 applicants (49%) submitted a fully completed survey.

#### Following the HTA guidance for applicants

All 47 applicants stated that they had referred to the NIHR HTA guidance for applicants when completing their application and ten of those 47 reported that they had followed the recommendation in the guidance to search the COMET database for a COS. Eight of these ten had not mentioned their search in their application, and of those eight, two found a relevant COS. Although they had referred to the guidance, a further eleven applicants stated that they did not search for a COS. Of the remaining 26 applicants, 19 reported that they had considered a COS using one or more resource other than the COMET database. Six considered a COS without a search of the COMET database as they were involved in the development of the COS, 12 carried out a search of the literature either as their only source of information or alongside other sources, seven applicants discussed the existence of COS with personal contacts and experts in the field, and it was not clear from seven applicants’ responses if they searched for a COS before deciding on their outcomes (Table 2). Ten of these 19 applicants that searched for a COS had not mentioned this in their application and one of those 10 found a COS.

**Table 2:** Survey responses of 47 applicants about searching for a COS.

	Searched COMET database	Involved in development of COS	Searched literature	Expert opinion	Did not search for a COS	Not clear if searched for a COS
No. of applicants*	10 (21%)	6 (13%)	12 (26%)	7 (15%)	11 (23%)	7 (15%)

* Some responders searched more than one source of information

#### Applicants’ decisions to use a COS

Applicants who found and used a COS, either through a search of the COMET database or some other source, provided a number of reasons for deciding to use the COS. Applicants referred to the benefits of COS (e.g. enabling comparison of studies and including outcomes that had been peer reviewed), external influence (e.g. from a Clinical Trials Unit and journal), and being involved in the development of a COS. Table 3 shows examples of applicants’ comments. Some applicants who found a COS that was relevant to the health condition in their study, even if not an exact fit for this, explained how they used the COS to inform their choice of outcomes. This included facilitating discussions of the applicants, experts and patient and public focus groups around which outcomes to choose, and incorporating some outcomes from the COS. Table 3 shows examples of applicants’ comments. In contrast, one applicant who found a COS that was relevant explained how they chose not to use it and instead used their experience of conducting trials and researching outcomes in the relevant health area to inform their choice of outcomes.

**Table 3:** Examples of reasons given by applicants for their decision to use a COS.

COS found and used	
Reason	Example
Benefits of using a COS	Essential to compare studies across the world
	Peer reviewed outcomes
**External influence**	Team in Clinical Trials Unit in… has been involved in the development of COS before and influenced my decision
	Journal publication requirements
Involved in the development of the COS	The COS for… was created from an… project—I was the lead investigator
	The lead author of the main COS publication was a co-applicant on the grant
**Relevant COS informed choice of outcomes**	
Facilitated discussions about outcomes	Yes the COS was used in discussions of choice of outcomes
	We also discussed the proposed outcome measures at a PPI focus group
	There was a great deal of discussion re the outcomes chosen with experts and PPI
Outcomes included in the trial	Core… outcomes incorporated
	Used the outcomes which were common in similar… research

#### Choice of outcomes by applicants not using a COS

Those applicants who responded to the survey and had already provided justification of choice of outcomes confirmed what they said in their applications. Additional information came from seven applicants who had not mentioned COMET or COS, or explained their choice of outcomes in their application. In the survey, these applicants echoed reasons given by others for their choice of outcomes, i.e. two were informed by a feasibility trial, six chose outcomes that had been used in other trials, five were informed by feedback from the funding board and three considered patient or public opinion.

## Discussion

Our study set out to assess the impact of a funder’s recommendation to clinical trialists to search for and use a COS in their study, and to discover how trialists choose outcomes to measure when a COS is not available. A number of trial funders endorse the use of COS and the COMET database (http://www.comet-initiative.org/cosuptake) and this study focused on the impact of the recommendation by the NIHR HTA programme.

Our results suggest that a funding body has the potential to have an impact on COS uptake by encouraging trialists to search for a COS. Based on the information provided by applicants in their application forms, and answers to survey questions, it is evident that at least 17 of 95 applicants searched the COMET database and seven of those applicants found a published COS to use in their trial or a COS in development to help inform their choice of outcomes. In addition another 19 applicants searched for COS using other sources, for example, a literature search, and 6 of those applicants found a published COS or a COS in development to inform their study. Out of a possible 24 applications that could have included a completed, published COS, seven (29%) applications did so.

However, it is possible that more applicants may have searched for and included a COS in their application but it was not possible to determine this in our assessment of the applications. This is because not all NIHR HTA applicants mention their search for a COS in their application form. The survey of Chief Investigators identified 18 applicants who had searched for a COS using the COMET database or another source but had not mentioned this in their application. Therefore, there may be more steps that could be taken by funding bodies beyond making the recommendation about COS that could further encourage uptake, make it possible to accurately assess the full impact of the recommendations, and ascertain whether the guidance is being adhered to.

For example, if the NIHR staff conducted their own search they might identify COS that should have been considered by the applicant. It would be useful for the funding board to be notified of the results of such a search, but it is important to acknowledge that this would require extra resources for staff to carry out the checks. For some funders this additional process may prove to be too resource intensive. A possible solution that would eliminate the need for additional resources in these circumstances might be for a member of the funding board to carry out a search of the COMET database during discussions about the outcomes included in the applications. It is evident from the application forms that 12 applicants in the cohort took advice about outcomes from the funding board and seven more applicants who had not mentioned this in their application forms reported having done so in the survey. If the board established whether a COS was available when discussing applications they could recommend its uptake in the feedback provided to the applicant. The search for a COS using the COMET database is a relatively quick process where a disease category or name can be selected and results can be restricted to show relevant COS for clinical trials. Although some applicants in our study used other ways to search for COS, e.g. by conducting their own search of the literature, the COMET database is recommended as it collates information about existing and developing COS in one place making it an effective resource for trialists. The content for the database comes from a systematic review[9] that is updated annually to ensure that the information held in the database is current[10–13].

As our study shows that some researchers are not explicit in their application about searching for COS, it may help to prompt them to report this by including a check list alongside the application form where the applicant can indicate what search they had done (e.g. of the COMET database). This would also provide further encouragement for applicants to search for a COS because although all survey responders stated that they used the guidance notes, eleven went on to report that they did not search for a COS. While it could result in extra burden for applicants to complete an additional checklist some journals have demonstrated that introducing such processes is possible by incorporating reporting guidelines as recommended by the EQUATOR Network (http://www.equator-network.org/toolkits/using-guidelines-in-journals/). This may include a requirement for authors to follow reporting guidelines and complete a checklist to confirm which guideline they have used that can be checked by editorial staff or peer reviewers. An alternative approach would be to include the recommendation about COS in the outcomes section of the application form, as well as in the guidance to applicants, to act as a further prompt to applicants. This approach has been put into practice by KCE, the Belgian Health Care Knowledge Centre (https://kce.fgov.be/en/kce-trials-2018-investigator-led-call). If the recommendation to search for and use COS became common practice across all funders of clinical trials, and the suggested processes for checking and further advising applicants were put in place, there would be great potential for funding bodies to have a significant impact on the uptake of COS in clinical trials.

Along with funders it is recognised that recommendations from other sources, such as trials registries, could facilitate the uptake of COS[14]. In addition, support from end users of COS, for example, clinical guideline organisations, HTA bodies and payers, may encourage uptake[15].

As well as the facilitators to COS uptake it is also important to consider the barriers. For example, one survey respondent in our study chose to use their team’s experience in trials and outcomes research to select outcomes for their study rather than using a COS. It is key that trialists are invested in a COS for their condition of interest for uptake to be achieved[16]. Without the investment of end users, the development of a COS is, as noted above, likely to result in research waste through poor uptake.

Although there are over 300 published COS available[13], there are still many conditions in need of a COS, and for 68 of the 95 applications assessed in this study a COS, or potentially relevant COS, did not exist at the time of submission. If those 68 applications were submitted now 32 would find a COS that has since been developed. Although the gaps are being filled, if a COS has not been developed, researchers need to access other sources to inform their decision about which outcomes to include in their trials and identification of the sources they use might highlight opportunities for COS developers to improve access to their work and its output. For example more than half the applicants included in this study sought the opinions of patients and the public on outcomes that should be included in their trial. This greater role for patients and the public supports the increasing focus on the inclusion of patients and the public in the development of COS[17, 18]. It also suggests that patients and the public could play an important role in the dissemination and implementation of COS.

Achieving full compliance by trialists to search for a COS when planning their trial is likely to take time to achieve as trialists become accustomed to the process, and it may require a change in culture for consideration of COS to become standard practice in clinical trials[15, 19]. As the number of COS continues to grow, and to allow time for trialists to adapt to searching for COS, we will endeavour to carry out another assessment of funding applications in three years’ time to determine if uptake of COS has increased. We recognise that future assessments should also include applications to other funders and not be limited to those submitted to the NIHR HTA. This will provide a wider view of the influence of funder recommendations on COS uptake.

To build on the findings of this study, we plan to conduct interviews with clinical trialists to solicit their opinions of COS and their development, and find out how trialists assess if a COS is relevant to their trial. This will allow a deeper understanding of the barriers that may limit, and facilitators that may encourage, uptake of COS.

To the best of our knowledge, this is the first study to assess the impact of a funder’s recommendation to search for and use COS on the choice of outcomes in applications for funding for clinical trials, building on efforts of funders to reduce research waste[20]. We analysed data from two sources (application forms and a survey of applicants) but acknowledge that that was limited to applications to a single funder, albeit the largest source of public funding for clinical trials in the UK. The survey included the need for the respondent to identify their project, which may have contributed to the proportion of non-respondents.

## Supporting information

S1 Appendix Data extraction matrix. (DOCX)

S1 Text Survey questions. (DOCX)

## Abbreviations

COMET: Core Outcome Measures in Effectiveness Trials
COS: Core outcome set(s)
EQUATOR: Enhancing the QUAlity and Transparency Of health Research
NIHR HTA: National Institute for Health Research Health Technology Assessment
