# Supplementary material for: Assessing the impact of a research funder’s recommendation to consider core outcome sets
Source: PLoS One. 2019 Sep 13;14(9):e0222418. doi: 10.1371/journal.pone.0222418 (PMC6743767; doi:10.1371/journal.pone.0222418)
Supplement: S1 Appendix — (DOCX) [file pone.0222418.s001.docx]

Data extraction matrix for researcher-led RCT applications

| **Funder ID** | **Date of funding decision** | **COMET database searched** | **Search for COS from other source** | **COS included in the study** | **If no search for COMET/COS (or no COS found) source accessed to inform outcome choice** | | | | | |
| --- | --- | --- | --- | --- | --- | --- | --- | --- | --- | --- |
|  |  |  |  |  | **PPI opinion** | **Outcomes used in other trials** | **Recommended by a professional body** | **Feedback from the funding board** | **Information from a pilot trial** | **Practitioner opinion** |
| xxx/xxx | xx/xx/xx | Y | N | Y |  |  |  |  |  |  |
| xxx/xxx | xx/xx/xx | Y | N | N | The patients have already approved our principal outcome measures |  |  |  |  |  |
| xxx/xxx | xx/xx/xx | N | Y | Y |  |  |  |  |  |  |
| xxx/xxx | xx/xx/xx | N | Y | N | The outcomes have been selected for being well developed, standardised measures with good . . . properties and which assess outcomes identified as important by patients. | Our decisions about which measures to use were informed by previous trials | Our decisions about which measures to use were informed by previous trials, . . ., and the . . . funded review of . . . outcome measures |  | We have also taken into account our own . . . feasibility study. |  |
| xxx/xxx | xx/xx/xx | N | N | N | Our primary endpoint is a clinical outcome (important and relevant to the patient and the . . .) |  |  | The primary outcome has been reviewed, reconsidered and refined to address the Board’s concerns. |  |  |
