# Supplementary material for: Assessing the impact of a research funder’s recommendation to consider core outcome sets
Source: PLoS One. 2019 Sep 13;14(9):e0222418. doi: 10.1371/journal.pone.0222418 (PMC6743767; doi:10.1371/journal.pone.0222418)
Supplement: S1 Text — (DOCX) [file pone.0222418.s002.docx]

**COS 1: Survey for applicants who had mentioned the COMET website or COS and had found and used a COS that had been published or was in development.**

1. What is your name please?
2. Please enter your project title or NETSCCID*

**HTA guidance notes for applicants**

1. When completing your HTA application did you refer to the guidance notes for applicants?*

No – (to question 4)

Yes – (to question 5)

1. If you did not refer to the guidance notes please select any of the following that apply*

- I have applied to the HTA funding scheme previously and was already aware of the application procedure
- I was not aware of the guidance notes for applicants
- Other – please specify

**Selection of outcomes for your HTA applications**

In January 2012, the NIHR HTA added the following statement to its guidance for applicants:

“Details should include justification of the use of outcome measures where a legitimate choice exists between alternatives.

- Where established Core Outcomes exist they should be included amongst the list of outcomes unless there is good reason to do otherwise. Please see The COMET Initiative website at [www.comet-initiative.org](http://www.comet-initiative.org) to identify whether Core Outcomes have been established.”

**We understand that you may have included a core outcome set (COS) in your application, or used a COS in development to inform your choice of outcomes**

1. How did you find out about the COS that you included in your application?*

Search of the COMET Initiative website

Search of the literature

Involved in the development of the COS

Other – please specify

1. What influenced your decision to use the COS?
2. Did you critically appraise the approach to COS development?

Yes – please got to question 8

No - please go to question 9

1. If yes, what factors did you consider?
2. Is there anything else that you would like to tell us about choosing outcomes for your study?
3. If you agree to be contacted about further studies relating to this PhD project please provide your email address:

Email:

**COS 2: Survey for applicants who had mentioned the COMET website or COS and had not found a relevant COS for their trial.**

1. What is your name please?
2. Please enter your project title or NETSCCID*

**HTA guidance notes for applicants**

1. When completing your HTA application did you refer to the guidance notes for applicants?*

No – (to question 4)

Yes – (to question 5)

1. If you did not refer to the guidance notes please select any of the following that apply*

- I have applied to the HTA funding scheme previously and was already aware of the application procedure
- I was not aware of the guidance notes for applicants
- Other – please specify

**Selection of outcomes for your HTA applications**

In January 2012, the NIHR HTA added the following statement to its guidance for applicants:

“Details should include justification of the use of outcome measures where a legitimate choice exists between alternatives.

- Where established Core Outcomes exist they should be included amongst the list of outcomes unless there is good reason to do otherwise. Please see The COMET Initiative website at [www.comet-initiative.org](http://www.comet-initiative.org) to identify whether Core Outcomes have been established.”

**We understand that you may have searched for a core outcome set (COS) but did not find one that was relevant to your study.**

1. Which of the following did you do to find out if a COS existed that was relevant to your study? Select all that apply*

Search of the COMET Initiative website

Search of the literature

Did not search for a COS

Other – please specify

1. Did you find a COS that may have been relevant to the health condition in your study even if it was not an exact fit for your trial?*

Yes – go to question 7

No – go to question 9

1. Did you use the COS to inform your choice of outcomes?*

Yes

No

1. Please give details*
2. Did any of the following influence your choice of outcomes?*

Patient and public involvement

Outcomes had been used in other trials

Informed by a feasibility trial

Feedback from the funding board

Other, please specify

1. Please explain how each of your answers above influenced your choice*
2. Is there anything else that you would like to tell us about choosing outcomes for your study?
3. If you agree to be contacted about further studies relating to this PhD project please provide your email address:

Email:

**COS 3: Survey for applicants who had not mentioned the COMET website or COS but had given reasons for their choice of outcomes.**

1. What is your name please?
2. Please enter your project title or NETSCCID*

**HTA guidance notes for applicants**

1. When completing your HTA application did you refer to the guidance notes for applicants?*

No – (to question 4)

Yes – (to question 5)

1. If you did not refer to the guidance notes please select any of the following that apply*

- I have applied to the HTA funding scheme previously and was already aware of the application procedure
- I was not aware of the guidance notes for applicants
- Other – please specify

**Selection of outcomes for your HTA applications**

In January 2012, the NIHR HTA added the following statement to its guidance for applicants:

“Details should include justification of the use of outcome measures where a legitimate choice exists between alternatives.

- Where established Core Outcomes exist they should be included amongst the list of outcomes unless there is good reason to do otherwise. Please see The COMET Initiative website at [www.comet-initiative.org](http://www.comet-initiative.org) to identify whether Core Outcomes have been established.”

1. Did you do any of the following to find out if a Core Outcome Set (COS) existed that was relevant to your study? Select all that apply.*

Search of the COMET Initiative website

Search of the literature

Did not search for a COS

Other – please specify

1. Did you find a COS that may have been relevant to the health condition in your study even if it was not an exact fit for your trial?*

Yes – go to question 7

No – go to question 9

1. Did you use the COS to inform your choice of outcomes?*

Yes

No

1. Please give details*
2. Is there anything else that you would like to tell us about choosing outcomes for your study?
3. If you agree to be contacted about further studies relating to this PhD project please provide your email address:

Email:

**COS 4: Survey for applicants who had not mentioned the COMET website or COS and did not give reasons for their choice of outcomes.**

1. What is your name please?
2. Please enter your project title or NETSCCID*

**HTA guidance notes for applicants**

1. When completing your HTA application did you refer to the guidance notes for applicants?*

No – (to question 4)

Yes – (to question 5)

1. If you did not refer to the guidance notes please select any of the following that apply*

- I have applied to the HTA funding scheme previously and was already aware of the application procedure
- I was not aware of the guidance notes for applicants
- Other – please specify

**Selection of outcomes for your HTA applications**

In January 2012, the NIHR HTA added the following statement to its guidance for applicants:

“Details should include justification of the use of outcome measures where a legitimate choice exists between alternatives.

- Where established Core Outcomes exist they should be included amongst the list of outcomes unless there is good reason to do otherwise. Please see The COMET Initiative website at [www.comet-initiative.org](http://www.comet-initiative.org) to identify whether Core Outcomes have been established.”

1. Did you do any of the following to find out if a Core Outcome Set (COS) existed that was relevant to your study? Select all that apply.*

Search of the COMET Initiative website

Search of the literature

Did not search for a COS

Other – please specify

1. Did you find a COS that may have been relevant to the health condition in your study even if it was not an exact fit for your trial?*

Yes – go to question 7

No – go to question 9

1. Did you use the COS to inform your choice of outcomes?*

Yes

No

1. Please give details*
2. Did any of the following influence your choice of outcomes?*

Patient and public involvement

Outcomes had been used in other trials

Informed by a feasibility trial

Feedback from the funding board

Other, please specify

1. Please explain how each of your answers above influenced your choice*
2. Is there anything else that you would like to tell us about choosing outcomes for your study?
3. If you agree to be contacted about further studies relating to this PhD project please provide your email address:

Email:
